# Association of anthropometric markers with globe position: A population-based MRI study

**DOI:** 10.1371/journal.pone.0211817

**Published:** 2019-02-07

**Authors:** Patrick Schmidt, Robert Kempin, Sönke Langner, Achim Beule, Stefan Kindler, Thomas Koppe, Henry Völzke, Till Ittermann, Clemens Jürgens, Frank Tost

**Affiliations:** 1 Department of Ophthalmology, University Medicine Greifswald, Greifswald, Mecklenburg-Western Pomerania, Germany; 2 Institute for Diagnostic Radiology and Neuroradiology, University Medicine Rostock, Rostock, Mecklenburg-Western Pomerania, Germany; 3 Department of Otorhinolaryngology, University Clinic Münster, Münster, North Rhine Westphalia, Germany; 4 Department of Oral and Maxillofacial Surgery/Plastic Surgery, University Medicine Greifswald, Greifswald, Mecklenburg-Western Pomerania, Germany; 5 Department of Anatomy and Cell Biology, University Medicine Greifswald, Greifswald, Mecklenburg-Western Pomerania, Germany; 6 Institute for Community Medicine, University Medicine Greifswald, Greifswald, Mecklenburg-Western Pomerania, Germany; National Institute of Public Health, MEXICO

## Abstract

**Purpose:**

Exophthalmometry is a common examination in ophthalmology. For example it is relevant for diagnosis or follow-up of thyroid eye disease. However, exophthalmometry is affected by several factors such as ethnicity, sex and age. The purpose of this study was to determine the globe position by magnetic resonance imaging (MRI) and to investigate its correlates among the general Northeast German adult population.

**Methods:**

A total of 3030 subjects aged between 20 and 89 from the population-based Study of Health in Pomerania (SHIP) underwent a standardised whole-body MRI. Axial length and globe position were determined in axial T1-weighted images of the orbit. The image had to include the corneal apex as well as the optic nerve head. Study participants were excluded from imaging analysis if there was no plane available that included both structures. Further exclusion criterion was a lateral deviation of the subject’s viewing direction. Images with inadequate quality due to motion artefacts or other technical reasons were excluded as well. Globe position was defined as the perpendicular distance between the interzygomatic line and the posterior surface of the cornea (exophthalmometric value). The distance between the posterior surface of the cornea and the posterior pole of the eyeball, at the boundary with orbital fat, was defined as axial length. We used posterior surface of the cornea for our measurements, because it seemed to be less vulnerable for motion artefacts than the anterior one. Moreover body measurements including body mass index (BMI), waist and hip circumferences were determined. Associations between anthropometric measurements with exophthalmometric outcomes were analysed by linear regressions adjusted for age and stratified by sex. P-values <0.05 were considered as statistically significant. To assess intra-reader variability intra-class correlation coefficients (ICC) were computed for repeated measurements of the MRI scans of 25 subjects.

**Results:**

After considering the exclusion criteria 1926 evaluable subjects remained. There was no significant difference between means of right and left eyes. The mean exophthalmometric value was significantly higher in men (16.5 +/- 2.2 mm) than in women (15.3 +/- 2.1 mm). The mean MRI-axial length was 23.4 +/- 0.8 mm for men and 22.8 +/- 0.9 mm for women. BMI, waist and hip circumferences were positively correlated with exophthalmometric value (p<0.001). Difference of mean MRI-based exophthalmometric value for obese subjects (BMI ≥30 kg/m^2^) and non-overweight (BMI <25 kg/m^2^) was 2.1 mm for men and 1.6 mm for women. ICC between 0.97 and 0.99 indicate excellent repeatability of our method.

**Conclusion:**

We conclude that MRI-based exophthalmometric values are positively correlated with BMI, waist- and hip-circumference among the general Northeast German adult population. This association is independent from age and axial length. Consequently bodyweight of patients should be regarded to interpret exophthalmometric values correctly. MRI-exophthalmometry seems to be a suitable method to determine globe position. Considering the large number of study participants, exophthalmometric values of our study could be used as comparative values for exophthalmometry of people of Western European descent in future.

## Introduction

Exophthalmometry is a routine examination in ophthalmology. Exophthalmometry quantifies the protrusion of the eyeball, resulting out of a discrepancy of orbital bony volume and the volume of its content. In clinical practice it is usually performed with an exophthalmometer. The most common one is Hertel’s exophthalmometer. The exophthalmometry is for example indispensable for diagnosis or follow-up of thyroid eye disease (TED), which is the most common reason for exophthalmos, due to an increase of orbital soft tissue volume. Multiple factors determine exophthalmometric values including ethnicity, sex and age [1, 2, 3, 4]. Probably there are even more parameters that may affect protrusion of the eyeball. It is of prime medical importance to detect these factors, so that physicians will be able to interpret individual exophthalmometric values of patients in a right way. The study of Smolders et al. indicated that the degree of ocular protrusion is related to a high body mass index (BMI) [5]. These results, however, were derived from a small study with only 64 subjects (19 obese/ 45 normal weight controls) [5]. In a population-based study among Sri Lankans, Chan et al. described a significant correlation of body weight and BMI with globe position [3]. Furthermore, Ibraheem et al. reported a significant association of body weight and BMI with protrusion of the eyeball in an African population [4]. They all used Hertel´s exophthalmometer to determine protrusion of the eyeball [3, 4, 5]. However, reliability of Hertel’s exophthalmometer is discussed controversially in literature [6, 7, 8]. Due to the great influence of ethnicity on the protrusion of the eyeball it is necessary that population-based studies concerning exophthalmometry are performed all over the world to get reliable comparables. Regarding central Europe there is no comparable study so far.

This study aimed to investigate whether exophthalmometric values are associated with BMI, waist and hip circumference in a population of German adults. In contrast to previous population-based studies that evaluated globe position, we used magnetic resonance imaging (MRI) for exophthalmometry. This method has only been applied on small cohorts so far [9, 10, 11].

## Materials and methods

Analyses are based on data from two independent cohorts of the Study of Health in Pomerania (SHIP). The study region of SHIP is West Pomerania, which is located in the Northeast of Germany. The aim of SHIP is to assess prevalence and incidence of common diseases and risk factors among the general population. In 1997 SHIP cohort was drawn by a two stage stratified cluster sample. Every five years a follow-up examination is conducted. Parallel to the second follow-up of SHIP (SHIP 2) an independent cohort was established between 2008 and 2012. This study cohort is called SHIP-Trend. For SHIP-Trend sample choice was simplified by using the local population registries of Mecklenburg-Western Pomerania. Therefore no two staged stratification process was needed. SHIP baseline and SHIP-Trend cohorts contained adults aged 20–79 years. Potential study participants were stratified by sex and age. The final sample was drawn at the proportional rate to the population of the particular place of residence. Eligible subjects were postally invited to participate in our study. In case of non-response they were contacted by telephone. The study population was 2333 for SHIP-2 and 4420 for SHIP-Trend. Detailed overviews about study designs, recruitment and procedures have been published previously [12, 13]. Whole-body MRI is a regular feature of SHIP-Trend and was also established in the second follow-up of SHIP (SHIP 2).

**Fig 1:**
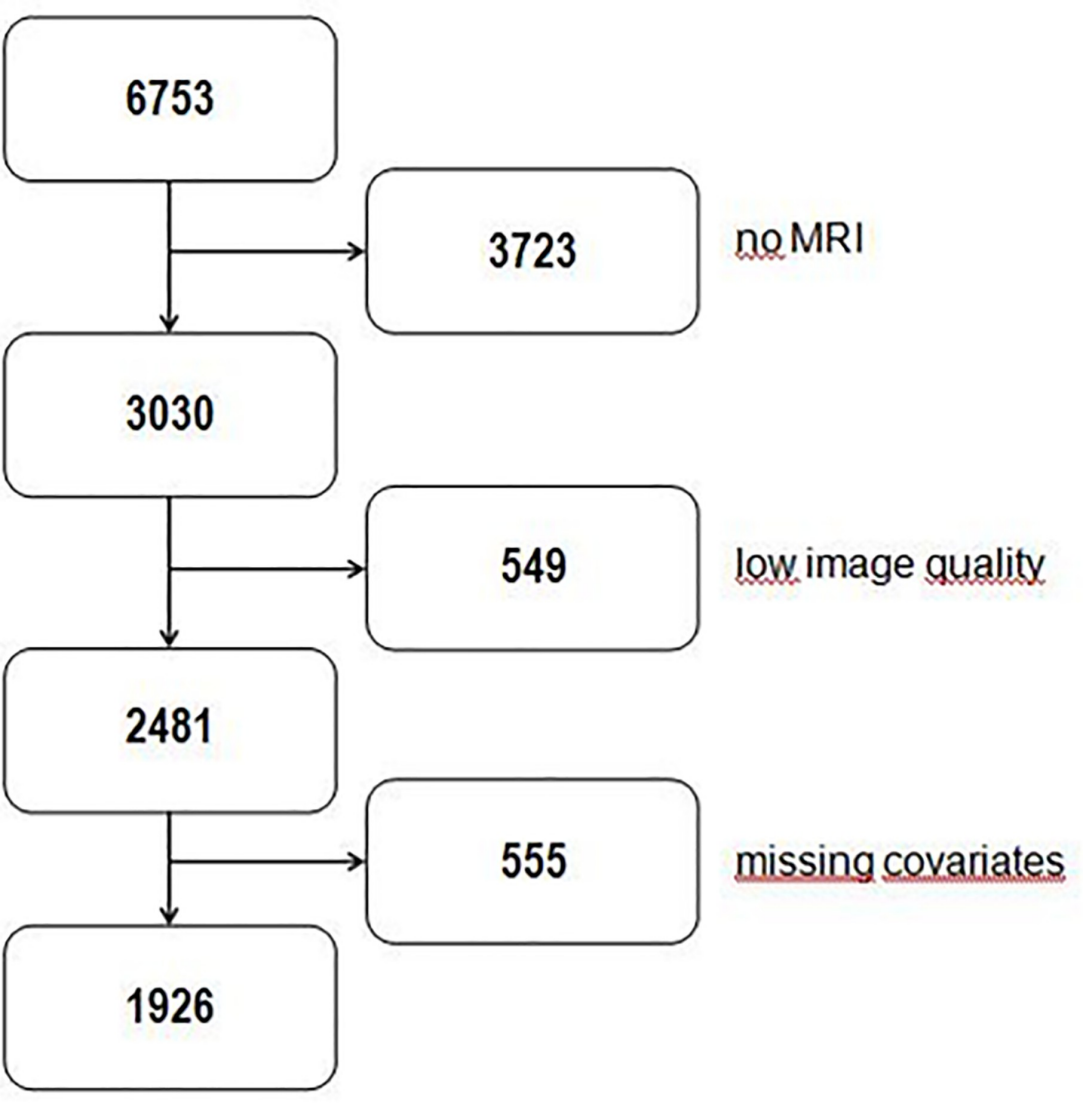
Flowchart describing the derivation of study samples for the analyses. 3723 study participants didn’t undergo MRI examination. 549 subjects were excluded due to low image quality or inadequate viewing direction (for detailed information see Materials and Methods). In some cases only one eye was evaluable or only axial length measurement but no exophthalmometry was possible. For this reason another 555 participants were excluded. After considering the exclusion criteria it was possible to determine axial length and exophthalmometry in 1926 cases for both eyes.

All 6753 subjects of both cohorts were invited to undergo whole-body MRI. Though 3723 study participants refused MRI examination or were excluded due to contraindications to MRI like metallic implants, tattoos or claustrophobia (see Fig 1). All whole-body MRI were acquired on a 1.5 T scanner Magnetom Avanto (Siemens Medical Solutions, Erlangen, Germany). Examination protocol was identical for all participants and has been published in detail previously [14]. In this study we used images without contrast media.

Data of 3030 individuals was available for our study. Subjects were aged between 20 and 89. All participants gave informed written consent. The study was approved by the Medical Ethics Committee of the University of Greifswald and followed the Declaration of Helsinki. All data of the study participants were accessed from an anonymised database. Axial T1-weighted images of the head were acquired using a 12-channel head coil. Images had a slice thickness of 1mm and the field of view was 256x256 mm. Other imaging parameters were TR 1900 ms, TI 1100 ms, TE 3.37 ms. During MRI examination, the subjects’ eyeballs rested in their spontaneous position without paying attention to a specific viewing direction. There was no guideline concerning eye lid position.

Image evaluation was performed with the Dicom-viewer OsiriX (Pixmeo, Geneva, Switzerland). For all MRI-measurements the image that bisected the eyeball in the axial plane was chosen. Zoom was set at 750%. Contrast was adjusted to a predefined W/L-setting of OsiriX by keying “0”to improve reliability of measurements. The image had to include the corneal apex as well as the optic nerve head. Consequently, false measurement due to Bell’s phenomenon could be avoided. Study participants were excluded from imaging analysis if there was no plane available that included both structures. Further exclusion criterion was a lateral deviation of the subject’s viewing direction. Images with inadequate quality due to motion artefacts or other technical reasons were excluded as well. Due to these exclusion criteria, which were summarized as “low image quality”, 549 subjects had to be precluded (see Fig 1). In some cases only one eye was evaluable or only axial length measurement but no exophthalmometry was possible. For this reason another 555 participants were excluded (see Fig 1). Consequently 1926 subjects were available for the present analysis. All measurements were performed by the same examiner (PS).

**Fig 2:**
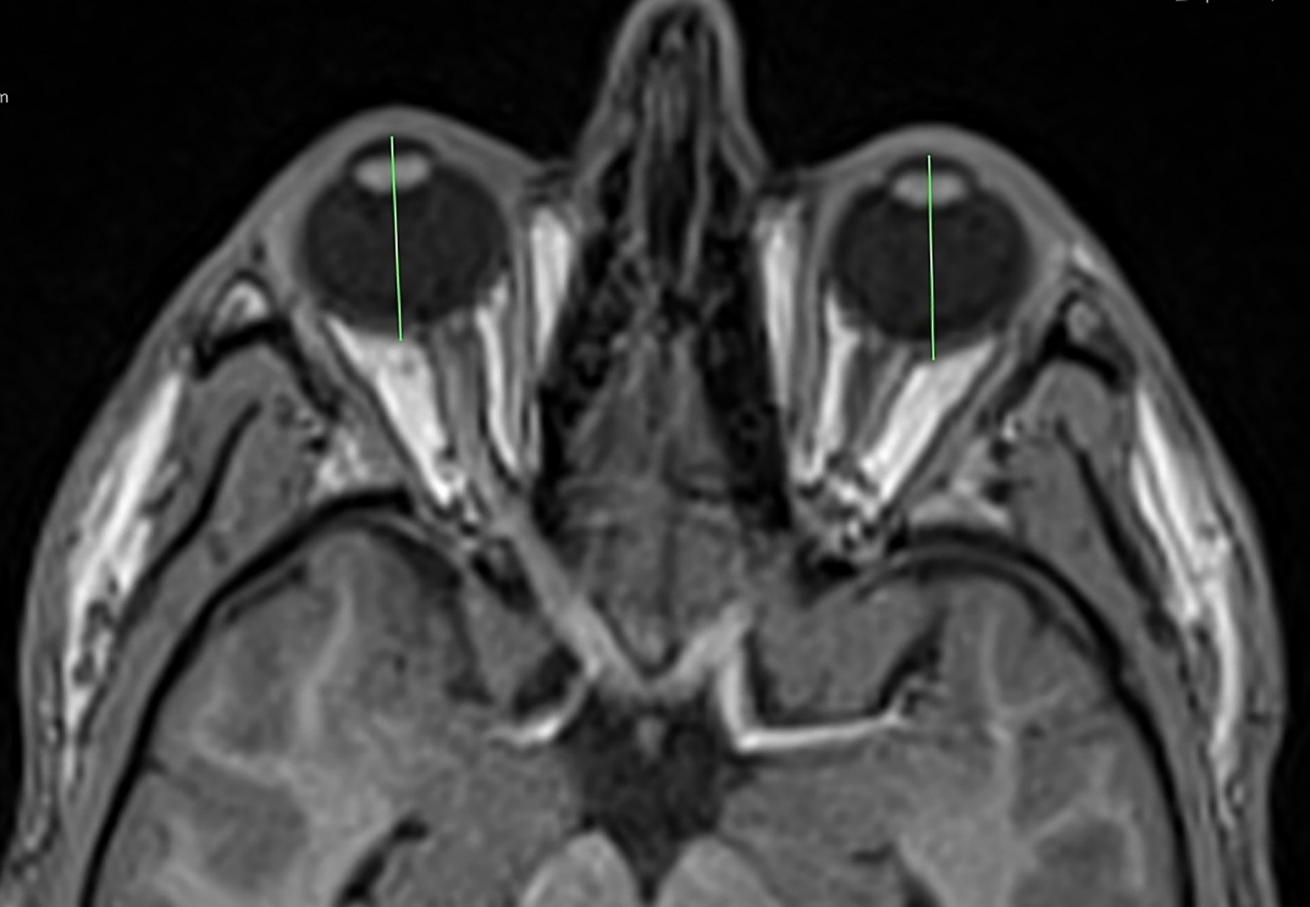
MRI-measurement of axial length.

**Fig 3:**
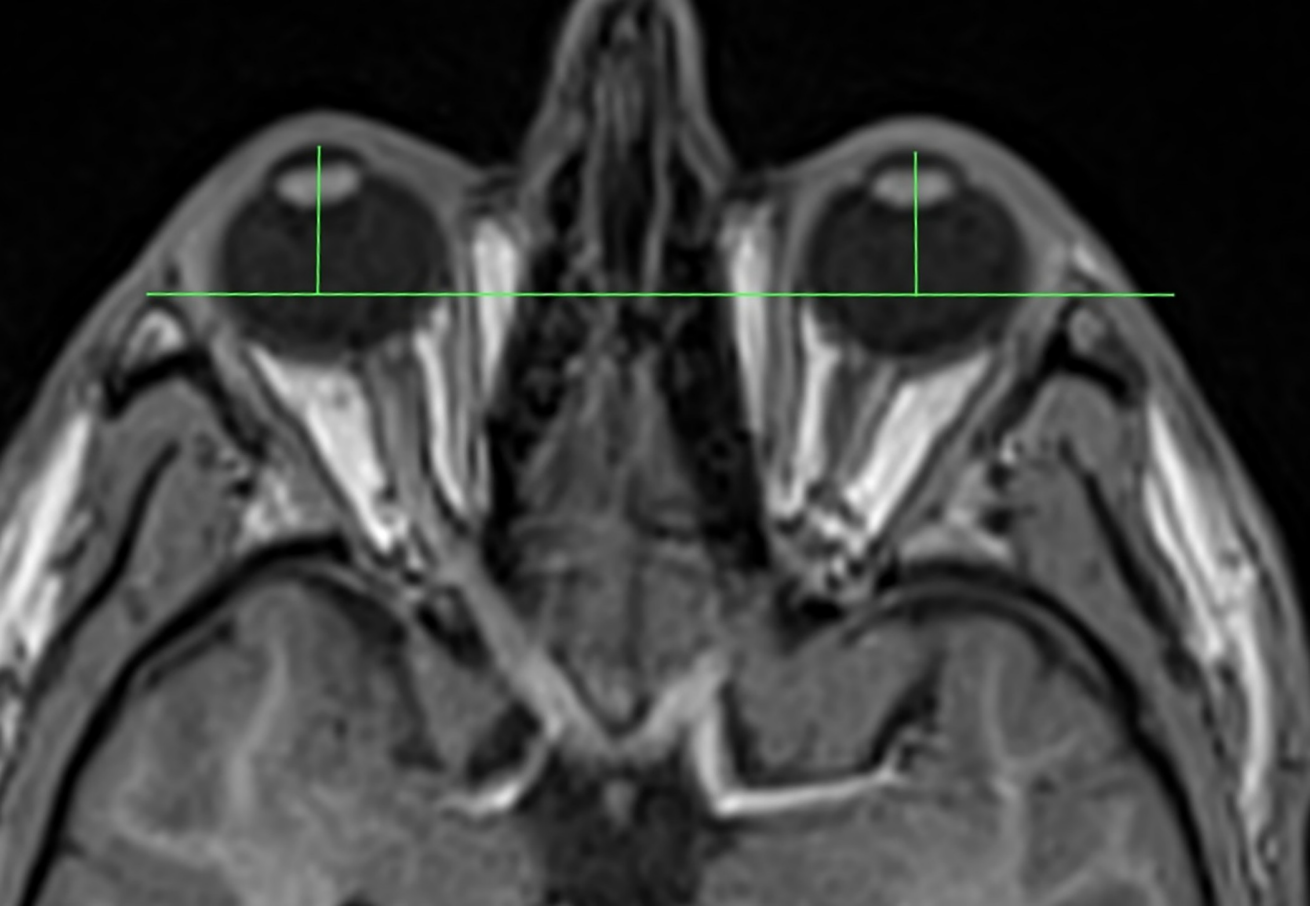
Technique for measuring proptosis.

MRI-axial length was measured from the posterior surface of the cornea to the posterior pole of the ocular bulb, at the boundary with orbital fat (see Fig 2). For MRI-exophthalmometry a line connecting the lateral orbital rims was used as reference (interzygomatic line). Cutis and subcutaneous fatty tissue were not considered. Exophthalmometric value was defined as the perpendicular distance between the interzygomatic line and the posterior surface of the cornea (see Fig 3). We used the posterior surface of the cornea for our measurements, because it seemed to be less prone for motion artefacts than the anterior surface. This technique has already been used for CT-exophthalmometry by Segni et al. [15].

Anthropometric measurements are part of the regular study protocol of SHIP and SHIP-Trend. We were able to access this dataset therefore no extra measurements were needed. To assess waist and hip circumference subjects had to stand in upright position. The examiner was placed lateral behind the study participant. The measuring tape always had to be horizontally. To determine waist circumference the examiner palpated the iliac crest and the lowest rip at the lateral part of the body. Measurements were made in the middle of these two landmarks. To assess hip circumference the examiner palpated the most lateral point of the greater trochanter and the iliac crest. Measurements were performed in the middle of these reference points. All body measurements were performed by trained study nurses considering a standard operating procedure (SOP).

To quantify the association between BMI and globe parameters, we divided our subjects into three different BMI-groups: non-overweight (BMI <25 kg/m^2^), overweight (25 kg/m^2^ ≤ BMI <30 kg/m^2^) and obese (BMI ≥30 kg/m^2^). The results are presented as mean and standard deviations for globe position and axial length.

Statistical analyses were performed using STATA 13.1 (STATA corp., College Station, USA). Continuous data are expressed as mean and standard deviations (SD); categorical data as absolute numbers and percentages. Associations between anthropometric measurements with exophthalmometric outcomes were investigated by linear regressions adjusted for age and stratified by sex.

To account for potential selection bias due to non-participation at the MRI examinations, inverse probability weights were calculated from a logistic regression model with participation as outcome and age, sex, smoking status, BMI, education and income as explanatory variables. These weights were used in all regression analyses. The aim of this approach was to weight up the influence of individuals from groups, which are more likely to drop-out. P-values <0.05 were considered as statistically significant.

To assess intra-reader variability MRI scans of 25 subjects were measured two times with a distance of several weeks between both evaluations and intra-class correlation coefficients (ICC) were computed for all globe parameters. ICC is a technique to evaluate the reliability of measurements or quantitative ratings. In statistics ICC assesses the reliability by comparing the variability of different parameters of subjects that are organized into groups and describes how strongly observations in the same group resemble each other. To illustrate the repeatability of our method we constructed Bland-Altman-plots.

## Results

After considering the exclusion criteria it was possible to determine axial length and exophthalmometry for both eyes in 1926 (1059 males/ 867 females) cases (see Fig 1). Mean age of the evaluable subjects was 52.7 +/- 14.3 years for males and 53.9 +/- 13 years for females. Mean BMI was 28.0 +/- 3.7 kg/m^2^ for males and 28.0 +/- 5.3 kg/m^2^ for females. Our study population consisted of 593 obese (309 males/ 284 females), 834 overweight (535 males/ 299 females) and 499 non-overweight (215males/ 284 females) subjects.

Waist circumference averaged 95.6 +/- 10.8 cm for men and 86.4 +/- 12.5 cm for women. Hip circumference of males (101.1 +/- 7.4 cm) and females (103.5 +/- 11.1 cm) was nearly equal. There was no statistically significant difference between mean values of right and left eyes. Mean MRI-based exophthalmometric value was significantly higher in men (16.5 +/- 2.2 mm) than in women (15.3 +/- 2.1 mm). Mean MRI-axial length was 23.4 +/- 0.8 mm for men and 22.8 +/- 0.9 mm for women (see Table 1).

**Table 1:** Description of the study population stratified by sex.

	Males (n = 1059)	Females (n = 867)
Axial length; mm (SD) right eye left eye Mean	23.4 (0.9) 23.4 (0.9) 23.4 (0.8)	22.8 (0.9) 22.8 (0.9) 22.8 (0.9)
Exophthalmometric value; mm (SD) right eye left eye Mean	16.6 (2.2) 16.5 (2.2) 16.5 (2.2)	15.3 (2.1) 15.2 (2.1) 15.3 (2.1)
Age; years (SD)	52.7 (14.3)	53.9 (13.0)
BMI; kg/m^2^ (SD)	28.0 (3.7)	28.0 (5.3)
BMI in categories (proportion) Normal weight Overweight Obesity	215 (20.3%) 535 (50.5%) 309 (29.2%)	284 (32.8%) 299 (34.5%) 284 (32.8%)
Waist circumference; cm	95.6 (10.8)	86.4 (12.5)
Hip circumference; cm	101.1 (7.4)	103.5 (11.1)

BMI = body mass index; SD = standard deviation.

The exophthalmometric value was significantly associated with axial length (p<0.001) and exophthalmometric value was positively correlated with BMI (p<0.001). By adjusting for age, sex and axial length we could show that the association of exophthalmometric value and BMI is independent of axial length. Waist and hip circumference were significantly associated with globe position (p<0.05). Per increase of one standard deviation for waist circumference mean exophthalmometric value ascended 0.91 mm for males and 0.78 mm for females (see Table 2). An increase of one standard deviation for hip circumference was associated with an augmentation of exophthalmometric values of 0.72 mm for males and 0.60 mm for females. Standard deviation was averaged for both sexes (12.5 cm for waist circumference and 9.3 cm for hip circumference). Mean exophthalmometric values of the different BMI groups are shown in Table 3. Discrepancy of mean exophthalmometric measurements for obese and non-overweight subjects was 2.1 mm for men and 1.6 mm for women.

**Table 2:** Sex-specific adjusted means for associations between age and mean ophthalmic parameters.

	Mean axial length; mm β (95%-CI)		Mean exophthalmometric value; mm β (95%-CI)	
	Males	Females	Males	Females
BMI (SD)	0.05 (-0.02; 0.11)	0.00 (-0.7; 0.05)	0.94 (0.76; 1.11)*	0.64 (0.51; 0.78)*
BMI in categories (SD) Non-overweight Overweight Obesity	Ref 0.14 (-0.02; 0.29) 0.14 (-0.02; 0.31)	Ref -0.06 (-0.21; 0.08) -0.05 (-0.21; 0.11)	Ref 0.95 (0.59; 1.32)* 2.04 (1.63; 2.44)*	Ref 0.73 (0.39; 1.07)* 1.66 (1.27; 2.05)*
Waist circumference (SD)	0.08 (0.01; 0.15)*	0.00 (-0.07; 0.08)	0.91 (0.75; 1.08)*	0.78 (0.62; 0.95)*
Hip circumference (SD)	0.15 (0.07; 0.23)*	0.01 (-0.04; 0.07)	0.72 (0.53;0.91)*	0.60 (0.47; 0.73)*

Results derived from linear regression adjusted for age; continuous values are standardized, so that the β’s describe the change in the outcome by 1 standard deviation (SD) of the exposure *p<0.05. CI = confidence interval; BMI = body mass index; Ref = reference.

**Table 3:** Mean exophthalmometric values and axial length for different BMI-groups.

BMI-group	sex	n	mean EV (mm)	mean axial length (mm)
**Non-overweight** (BMI <25 kg/m^2^)	men	215	15.4	23.4
	women	284	14.5	22.8
**overweight** (25 kg/m^2^ ≤ BMI <30 kg/m^2^)	men	535	16.4	23.4
	women	299	15.2	22.7
**obese** (BMI ≥30 kg/m^2^)	men	309	17.5	23.4
	women	284	16.1	22.8

Mean exophthalmometric values and axial length for different BMI-groups. BMI = body mass index; EV = exophthalmometric values

The augmentation of exophthalmometric values per increase of one standard deviation for BMI was also more pronounced among male subjects (0.94 mm for men and 0.64 mm for women). There was no association between axial length and anthropometric markers, besides a weak correlation of waist and hip circumference with axial length in males (see Table 2).

**Fig 4:**
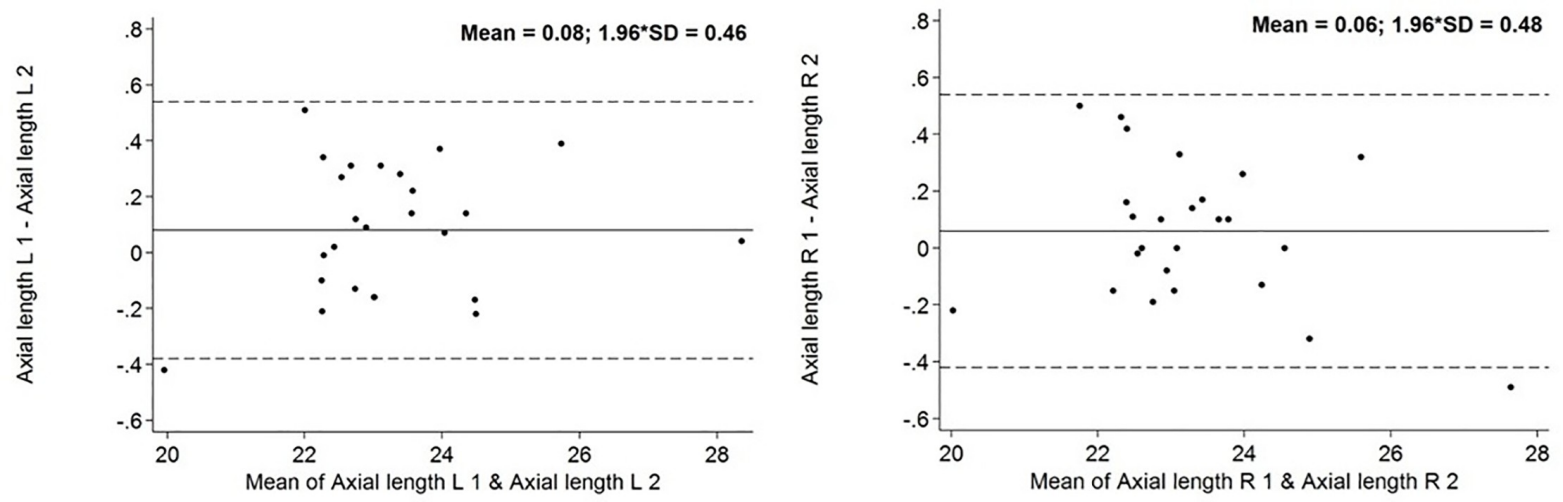
Bland-Altman Plot concerning MRI-axial length. Solid line is the mean difference, and the dotted lines are mean difference − SD (lower) and mean difference + SD. The points represent the individual deviation of each remeasurement compared with the original measurement. Axial length in mm.

**Fig 5:**
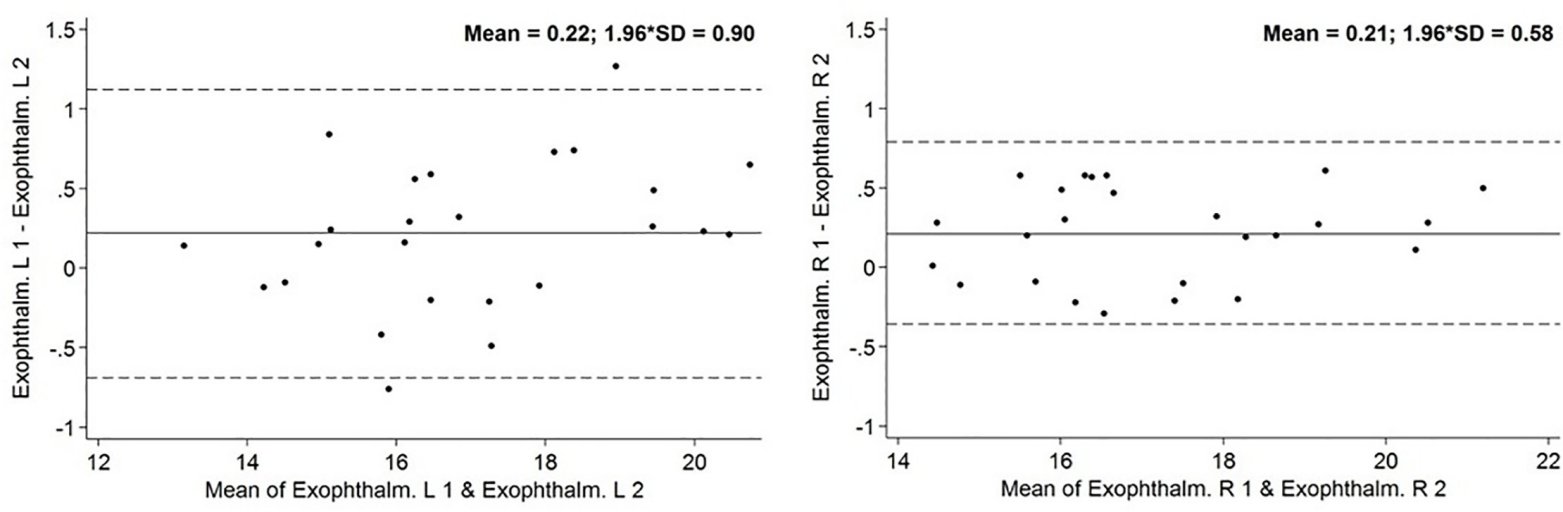
Bland-Altman Plot concerning MRI-exophthalmometry. Solid line is the mean difference, and the dotted lines are mean difference − SD (lower) and mean difference + SD. The points represent the individual deviation of each remeasurement compared with the original measurement. Exophthalmometry in mm.

ICC for intra-reader variability of axial length measurements and exophthalmometry were between 0.97 and 0.99. The Bland-Altman-Plots diagram the deviation of remeasurements compared with the values of the first one. Bland-Altman Plots are presented in Figs 4 and 5.

## Discussion

Exophthalmometry is a frequent examination in ophthalmology. However, exophthalmometric values are influenced by diverse factors such as ethnicity, age and sex [1, 2, 3, 4]. It is important to detect these factors, so that physicians will be able to interpret individual exophthalmometric values of patients correctly in future. In clinical practice exophthalmometry is usually performed with the aid of an exophthalmometer. The most common one is Hertel’s exophthalmometer. Nevertheless the reliability of this method is discussed controversially in literature [6, 7, 8].

While Bingham et al. reported an excellent intra- (ICC 0.98) and interobserver (ICC 0.91 and more) reproducibility for Hertel’s exophthalmometer, the results of Sleep et al. question the reliability of this method [6, 8]. Moreover Frueh et al. expounded the error-proneness of Hertel’s exophthalmometry [7]. Measuring error of Hertel’s exophthalmometer is potentially caused by parallax. Therefore, Mourits developed the so called parallex-free Mourits exophthalmometer to minimize this source of error. Bingham et al. compared measurements of Hertel, Oculus and Mourits exophthalmometers [6]. They could not show an advantage of the parallex-free Mourits exophthalmometer [6]. On the other hand Genders et al. as well as Delmas et al. indicated good reliability for Mourits exophthalmometer [16, 17]. Further investigation is needed to prove the superiority of Mourits exophthalmometer regarding intra and interobserver reliability. Another approach to assess globe position is the exophthalmometer by Naugle. It uses superior and inferior orbital rim as reference. Cole et al. did not report significant differences of exophthalmometric values measured by Hertel and Naugle instruments [18]. Due to the unique concept of SHIP, implying standardized whole body MRI of non-hospitalized volunteers, this study is able to report population-based, representative, MRI-based values of globe position and its association to anthropometric measurements for a cohort of people of predominantly European descent for the first time. The results of this study demonstrate that MRI-based exophthalmometric values are associated with BMI, waist- and hip-circumference among the general Northeast German population. Mean values of the different BMI groups clarify the great influence of anthropometric markers on the protrusion of the eyeball. Besides this study is the first to report that the correlation between BMI and exophthalmometric value is independent from age, sex and axial length of the globe. ICCs between 0.97 and 0.99 indicate an excellent repeatability of the developed method.

The results of Smolders et al, Ibraheem et al. and Chan et al. are consistent with our findings [3, 4, 5]. In contrast to our study they all used Hertel's exophthalmometer to determine protrusion of the eyeball [3, 4, 5]. Smolders et al. defined different BMI groups and compared the mean exophthalmometric values [5]. Discrepancy of mean protrusion for obese (BMI ≥30 kg/m^2^) and non-obese (26 kg/m^2^ > BMI >20 kg/m^2^) subjects of European descent was about 5 mm [5]. In our study difference of mean exophthalmometric values for obese (BMI ≥30 kg/m^2^) and non-overweight subjects (BMI <25 kg/m^2^) was smaller (2.1 mm for men and 1.6 mm for women). However Smolders et al. examined only 64 subjects without any population-based selection procedure [5]. The group of obese patients in the study of Smolders et al. counted 8 males and 11 females [5]. In contrast, we analysed Data of 1926 volunteers which were randomly recruited among the general population. Moreover the average BMI in the obese group (36.5 kg/m^2^ for females and 39.0 kg/m^2^ for males) of Smolders et al. was higher than in this study (32.5 +/- 2.2 kg/m^2^ for men and 34.1 +/- 3.5 kg/m^2^ for women) [5]. Due to the great discrepancy of mean globe position, standard values for exophthalmometry should be used with caution for obese individuals. Mean exophthalmometric values of the different BMI-groups in our study may be useful to assess globe position in obese patients. Nevertheless, it should be mentioned that values of MRI-exophthalmometry are not completely comparable to measurements with an exophthalmometer. Our method uses posterior margin of the cornea as reference and does not consider cutis and subcutaneous fatty tissue. Pre zygomatic arc fat tissue is a possible source of bias because the difference between exophthalmometric measurements with Hertel and MRI might be higher in obese subjects.

However, the study of Smolders et al. clarify an association between BMI and exophthalmometry, despite measurements were performed by Hertel’s exophthalmometer which regards, in contrast to our method, subcutaneous fatty tissue [5]. Consequently obesity should always be considered as a potential cause for exophthalmos. Therefore increasing volume of orbital fat and fatty infiltration of eye muscles are discussed as possible reasons for high exophthalmometric values in obese subjects [5]. The association between BMI and exophthalmometric values was more pronounced among men (The mean difference of exophthalmometric values for obese and non-overweight subjects was 2.1 mm for males and 1.6 mm for females. Per increase of one standard deviation for BMI mean exophthalmometric value ascended 0.94 mm for males and 0.64 mm for females). The amount of non-overweight subjects was smaller in the male subpopulation (n = 215~20.3%) than in the female group (n = 284~32.8%). Otherwise the size of the obese groups was nearly equal for both sexes (n = 309~29.2% men and n = 284~32.8% women) (see Table 1). In addition mean age and BMI in obese (men: BMI: 32.5 kg/m^2^, age: 55.1 years/women: 34.1 kg/m^2^, age: 57.2 years) and non-overweight (men: BMI: 23.2 kg/m^2^, age: 45.9 years/ women: BMI: 22.6 kg/m^2^, age: 48.7 years) groups were comparable for both sexes. However, diverse fat distribution and orbital bone anatomy could be possible reasons for the discrepancy. This assumption is supported by publications which reported a significant variation for orbital volume or orbital soft tissue volume in males and females [19, 20, 21].

To our knowledge the relation between waist and hip circumference with exophthalmometric values has never been investigated before. Our results demonstrate that globe position is related to these anthropometric markers.

As with BMI the association between these two markers and exophthalmometric values is more pronounced among male study participants.

MRI-exophthalmometry has only been applied on small samples so far [9, 10, 11]. Consequently there has been no standardized procedure for MRI-exophthalmometry so far. Because closed eyelids were often indistinguishable from the anterior surface of the cornea or motion artefacts blurred the edge of the corneal surface, we used the posterior margin of the cornea as reference for our measurements. This technique has already been used for CT-exophthalmometry by Segni et al. [15]. Segni et al. could show an excellent reproducibility for this measurement technique (ICC0.988 for repeated measurements) [15]. However, exophthalmometric values tended to be lower than measured by exophthalmometer [15]. It is important to keep this fact in mind when you want to compare exophthalmometric values measured by these different techniques. Consequently, inter-technique comparisons of absolute values can be inaccurate and should be avoided [6, 15]. In addition other studies have also evaluated reliability of CT-exophthalmometry [6, 22]. Nevertheless, they used anterior surface of the cornea as reference [6, 22]. Ramli et al. reported excellent intra-observer reproducibility (ICC 0.99) [22]. Bingham et al. also evaluated a brilliant inter-observer agreement (ICC >0.99) [6]. Because of the higher radiographic contrast of soft tissue MRI seems to be more suitable for exophthalmometry than CT. But this method has only been applied in a few studies on small cohorts so far [9, 10, 11]. There is no information about inter- or intra-reader variability of MRI-exophthalmometry available in literature so far. In our study intra-reader variation was assessed by repeated measurements of MR-images by the same examiner.

ICC between 0.97 and 0.99 indicate excellent repeatability of our method. This excellent repeatability is graphically represented in Bland-Altman-Plots (Figs 4 and 5). The excellent repeatability of our method emphasizes the relevance of our findings. Like in our study, Detorakis et al. as well as Ozgen et al. used a line connecting the lateral orbital rims (interzygomatic line) as reference for exophthalmometry [9, 10, 11]. Detorakis et al. defined protrusion of the eyeball as the perpendicular distance between the reference line and the corneal apex in axial images [9]. Detorakis et al. analysed DATA of 46 subjects (28 men/ 18 women) [9]. They also used T1 weighted images with a slice thickness of 1 mm, acquired by a 1.5 T MRI system [9]. Dektorakis et al. reported higher exophthalmometric values in women (16 mm) than in men (14 mm) [9]. However, remeasurements with Hertel’s exophthalmometer show higher values for male subjects (16 mm/ women 15 mm) [9]. Ozgen et al. chose another method to determine protrusion of the eyeball [11]. They used the distance between the interzygomatic line and the posterior pole of the eyeball to describe globe position [11]. Ozgen et al. analysed axial T1 weighted images acquired by a 0.5 T MRI system with 3 mm thick sections [11]. Ozgen et al. evaluated DATA of 100 subjects with a mean age of 41 (44 men/ 56 women) [11]. The mean distance between the interzygomatic line and the posterior pole of the eyeball was 8.9 mm, without any statistically significant difference of males and females [11]. However, this method has a crucial disadvantage. Using the posterior margin of the eyeball to describe globe position makes it more difficult to compare the results with routine examinations by exophthalmometer. Both Ozgen et al. as well as Detorakis et al. did not define a precise image plane to perform MRI-exophthalmometry in axial images [9, 10, 11]. Without determining a precise plane for MRI-exophthalmometry, measurements are sensitive to errors.

In our view MRI-exophthalmometry should only be performed in image planes including the corneal apex as well as the optic nerve head to avoid measurement errors. We suggest to observe these guidelines in future CT- or MRI-exophthalmometries to make measurements more reliable and comparable. We consider that MRI-exophthalmometry seems to be a suitable method to determine globe position. In contrast to CT-exophthalmometry there was no radiation exposure for our study participants, which is of superior ethical importance in the concept of an epidemiological study, such as SHIP. A limitation of our study is the considerable proportion of excluded study participants. In the future acquiring of images for MRI-exophthalmometry should only be performed in standardized head position. Maybe fixation of the head similar to techniques of radiation therapy would be helpful. Additionally the study participants should keep their sight rigorously forward to prevent motion artifacts. Eyelids should not be closed to avoid Bell’s phenomenon. Cause in case of Bell’s phenomenon cornea and optic nerve head are not part of the same MRI image. Consequently, MRI-exophthalmometry or axial length measurement could not be performed or would be erroneous. TED which was not assessed in our study might also be a potential source of variation. However, the prevalence of TED was calculated to be 0.25% and therefore should not affect the detected associations in our analysis [23]. Moreover MRI-exophthalmometry is expensive in contrast to measurements with exophthalmometers. However, in cases with exophthalmos of unknown origin an imaging of orbital structures is indispensable. In these cases standard MRI-exophthalmometry had great relevance and would not occasion any extra costs. Besides exophthalmometry measurement the cause of the protrusion, like soft tissue enlargement or fatty infiltration of eye muscles in the course of TED, could be detected within the same examination.

We conclude that exophthalmometric values are positively correlated with BMI, waist- and hip-circumference among the general Northeast population of Germany. This association is independent from age and axial length. Because of that bodyweight of patients should be regarded to interpret exophthalmometric values correctly. MRI-exophthalmometry seems to be a proper way to determine globe position. Considering the large number of study participants, exophthalmometric values of our study could be used as comparative values for MRI-exophthalmometry of people of European descent in future.
